# Two Mechanisms Regulate Keratin K15 Expression In Keratinocytes: Role of PKC/AP-1 and FOXM1 Mediated Signalling

**DOI:** 10.1371/journal.pone.0038599

**Published:** 2012-06-27

**Authors:** Amrita Bose, Muy-Teck Teh, Iain L. Hutchison, Hong Wan, Irene M. Leigh, Ahmad Waseem

**Affiliations:** 1 Centre for Clinical and Diagnostic Oral Sciences, Institute of Dentistry, Barts and The London School of Medicine and Dentistry, Queen Mary University of London, London, United Kingdom; 2 Division of Cancer, Medical Research Institute, University of Dundee, Dundee, United Kingdom; Virginia Commonwealth University, United States of America

## Abstract

**Background:**

Keratin 15 (K15) is a type I keratin that is used as a marker of stem cells. Its expression is restricted to the basal layer of stratified epithelia, and the bulge in hair follicles. However, in certain clinical situations including oral lichen planus, K15 is induced in suprabasal layers, which is inconsistent with the role of a stem cell marker. This study provides insights into the mechanisms of K15 expression in the basal and differentiating keratinocytes.

**Methodology/Principal Findings:**

Human keratinocytes were differentiated by three different methods; suspension in methylcellulose, high cell density and treatment with phorbol ester. The expression of mRNA was determined by quantitative PCR and protein by western blotting and immunostaining. Keratinocytes in suspension suppressed β1-integrin expression, induced differentiation-specific markers and K15, whereas FOXM1 (a cell cycle regulated protein) and K14 were downregulated. Rescuing β1-integrin by either fibronectin or the arginine-glycine-aspartate peptide suppressed K15 but induced K14 and FOXM1 expression. Specific inhibition of PKCδ, by siRNA, and AP-1 transcription factor, by TAM67 (dominant negative c-Jun), suppressed K15 expression, suggesting that PKC/AP-1 pathway plays a role in the differentiation-specific expression of K15. The basal cell-specific K15 expression may involve FOXM1 because ectopic expression of the latter is known to induce K15. Using chromatin immunoprecipitation, we have identified a single FOXM1 binding motif in the K15 promoter.

**Conclusions/Significance:**

The data suggests that K15 is induced during terminal differentiation mediated by the down regulation of β1-integrin. However, this cannot be the mechanism of basal/stem cell-specific K15 expression in stratified epithelia, because basal keratinocytes do not undergo terminal differentiation. We propose that there are two mechanisms regulating K15 expression in stratified epithelia; differentiation-specific involving PKC/AP-1 pathway, and basal-specific mediated by FOXM1, and therefore the use of K15 expression as a marker of stem cells must be viewed with caution.

## Introduction

In the epidermis, the basal keratinocytes are unique due to their attachment to the basement membrane and they constitute the major proliferating cell population. Some of the basal keratinocytes are stem cells that are characterised by their longevity and clonogenicity. These cells are responsible for tissue homeostasis and regeneration of epidermis following injury [1], [2], [3], [4], [5], [6]. As the stem cells divide, they produce transit-amplifying (TA) cells, which provide a constant supply of ‘committed’ cells to replenish those lost during differentiation (reviewed in [7]). The committed keratinocytes downregulate integrins to become less adhesive, move to the suprabasal compartment and continue their upward movement until they are terminally differentiated [8] and shed off. This produces several layers of keratinocytes, at different stages of differentiation, which provide a protective barrier. The different layers of epidermis can be identified by the expression of keratins, which are a very large family of proteins (49 genes in the human genome [9]) that form heteropolymers of type I (acidic) and type II (basic/neutral) polypeptides in ‘soft’ and ‘hard’ epithelia [10]. A member of each type is necessary to form filaments and the pairwise expression is highly differentiation specific. For example, the basal keratinocytes express keratins K5, K14 and K15, whereas differentiating keratinocytes in the epidermis express keratins K1 and K10 [11], [12] and in the mucosae they express keratins K4 and K13 [13]. K6, K16, K9 and K17 are expressed in palmoplantar epidermis [14] and also in psoriasis, hypertrophic and keloid scars and in some epidermal tumours [15], [16]. The transcription of basal keratins is switched off as soon as the basal keratinocytes move into the suprabasal compartment with concomitant induction of differentiation-specific keratins [11], [17].

K15 is a type I keratin which does not have its own type II expression partner for filament assembly and shares the type II partner, K5, with K14 [18]. This protein attracted the investigators’ attention much later because it was a minor component of the epidermis, and it had a molecular weight similar to K14, so it could only be separated by 2-D gel electrophoresis [11], [19]. The first polyclonal antibody against K15 was described in 1995/96 for tissue expression studies [18], [20]. Later work used C8/144B, LHK15 and a cross reacting LC18N monoclonal antibodies, which established that K15 was specifically expressed in the basal keratinocytes of most stratified epithelia [21], [22], [23].

In the epidermis, K15 expression is discontinuous in adults, with strongest staining in rete-ridges and weakest in the dermal papillae [21], [24]. There are several lines of evidence to suggest that K15 is a marker of stem cells. First, K15 is strongly expressed in the bulge compared with the rest of the follicle [21], [23], [25]. Second, K15 expression has been used to detect limbal stem cells on ocular surfaces [26]. Third, K15+ keratinocytes express lower levels of the pro-apoptotic gene Bax, but higher levels of anti-apoptotic genes than the K15- cells, which is consistent with their stem cell nature [24]. Fourth, K15 promoter was able to target β-galactosidase to the bulge [27] and bulge cells expressing K15 promoter driven GFP reconstituted entire epidermis and had higher proliferation potential [28]. Fifth, the Lgr5+ cells in the bulge, capable of regenerating the entire follicle, were K15+ [29]. These observations are inconsistent with other reports that do not support K15 as a marker of stem cells. For example, in sheep hair follicles K15 is expressed in the outer root sheath except in the bulge [30]. Furthermore, in mucosal epithelia, the K15 expression is uninterrupted throughout the basal layer and it is unlikely that every basal cell would be a stem cell. This may indicate that in mucosae either the stem cell zone has expanded or it is heterogeneous containing stem and non-stem cells as in the bulge [29], [31]. Nevertheless, this indicates that K15 expression shows variation in different epithelia in a species-specific fashion.

The expression of K15 has been reported to change in a large number of human diseases, for example, K15 is increased in basal cell carcinoma (BCC) but it is suppressed in squamous cell carcinoma [22], [32], [33], [34]. In hyperproliferating conditions, such as during wound healing, pathological scarring and also in some psoriatic samples, the K15 expression is suppressed, suggesting that the cellular environment inside activated keratinocytes may not be conducive to K15 expression [21], [22], [35]. The downregulation of K15 in inflammatory conditions is consistent with the reported suppression of K15 transcription by activation-specific growth factors and cytokines including the transforming growth factor-β, the tumour necrosis factor-α, the epidermal growth factor and keratinocyte growth factor [36]. These reports also suggest that K15 may not be a reliable stem cell marker on its own, especially in diseased epithelia [35], [37].

The regulation of K15 is more complex than other keratins and appears to depend on the presence of other keratins, especially the level of K14. For example, increased expression of K15 has been observed in K14 knockout mice and also in EBS patients lacking K14 [18], [20]. The level of K15 expression and its localisation in the basal layer has also been reported to change with age [18], [38]. In certain stratified epithelia, such as the oesophagus, and in certain clinical situations, such as oral lichen planus, K15 is expressed in the suprabasal layers [22], [39], [40]. This, together with the reported induction of K15 synthesis in keratinocytes at high cell densities [22], [41], suggests that K15 can also be expressed in differentiating keratinocytes. In a detailed analysis of the K15 promoter (1250 bp upstream region), Radoja and co-workers showed that C/EBP-β, AP-1, thyroid hormone and IFN-γ induced the K15 promoter, whereas retinoic acid, glucocorticoid receptors, and NF-κB suppressed it [42]. These studies, although highly informative, do not explain the mechanism of basal and suprabasal expression of K15 in stratified epithelia. No study thus far has investigated the molecular mechanism regulating the differentiation-specific expression of K15. Here we provide the first mechanistic evidence that K15 expression could be induced by differentiation through PKCδ and AP-1. We also show that the basal cell (or stem cell) specific expression of K15 involves a different mechanism, perhaps mediated by the cell cycle regulated transcription factor FOXM1. This study will help resolve conflicting data in the literature on the expression of K15 and cautions to its use as a marker of stem cells.

## Results

### Induction of Keratin K15 in Differentiating Human Keratinocytes

**Figure 1:**
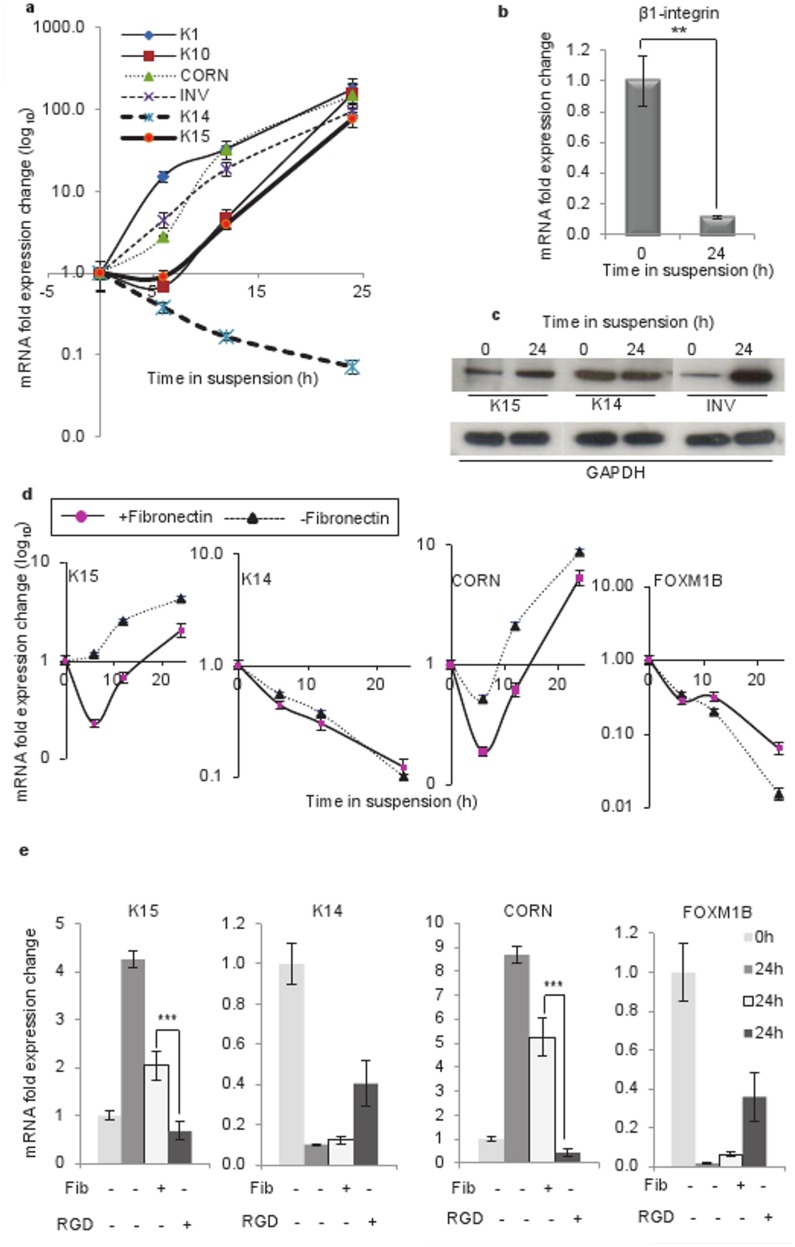
Induction of K15 expression in keratinocytes suspended in methylcellulose solution. (a) N-Terts growing in SFM were suspended in DMEM +20% FCS containing 1.3% (w/v) methylcellulose. At different time, total mRNA was isolated, converted into cDNA and used to determine the expression of K1, K10, cornifin, involucrin, K14 and K15 by real-time absolute qPCR. (b) Downregulation of β1-integrin following 24 h incubation of N-Terts in methylcellulose. (c) Expression of K15, K14 and involucrin protein by western blotting following incubation of N-Terts in suspension at 0 and 24 h. (d) Influence of 100 µg/ml fibronectin on the time course of keratinocyte differentiation as measured by the expression of K15, K14, cornifin and FOXM1B. (e) Comparison of fibronectin (Fib) and RGD peptide (RGD) on gene transcription in suspension culture at 0 and 24 h. The 0 h cells were used for mRNA extraction soon after trypsinisation before being suspended in methylcellulose. Each bar represents the mean±SEM where n = 3. (P<0.01, very significant, **; P<0.001, extremely significant, ***).

**Figure 2:**
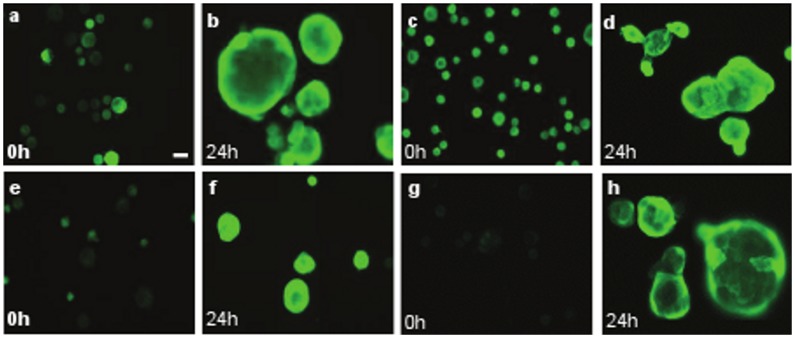
Induction of K15 in terminally differentiated N-Tert keratinocytes. N-Terts growing in SFM were suspended in DMEM +20% FCS containing 1.3% (w/v) methylcellulose. At 24 h the cells were harvested and washed with PBS before being suspended in 20–30 µl FCS. A 3–4 µl aliquot was streaked on glass slides. At the same time freshly trypsinised N-Terts were also streaked and used as 0 h control. The cells were dried in air, fixed in formaldehyde and immunostained with different monoclonal antibodies as described in the ‘Materials and Methods’ section. K15 (a, b), K14 (c, d), Involucrin (e, f), Cornifin (g, h). All slides were photographed at the same magnification. The cells in b, d, f and h were difficult to focus because they underwent aggregation in methylcellulose (magnification bar  = 20 µm).

To study keratin gene regulation during differentiation, we suspended keratinocytes (N-Terts) in 1.3% (w/v) methylcellulose and we were expecting a downregulation of K14 and K15 genes because both were basal-specific keratins. However, to our surprise we observed a time-dependent downregulation of K14 but an upregulation of K15 gene transcription (Fig. 1a). To demonstrate that keratinocytes were indeed undergoing differentiation in methylcellulose, we measured a series of differentiation-specific genes K1, K10, cornifin and involucrin. As shown in figure 1a, the transcription of differentiation-specific genes increased with time and reached a maximum at 24 h. The transcriptional induction of K10 and K15 displayed a lag phase, which was absent in other genes (Fig. 1a), indicating that their induction perhaps required synthesis of additional factors. Methylcellulose suspension is known to induce terminal differentiation by downregulating β1-integrin [8], [43]. To demonstrate that keratinocyte differentiation was indeed mediated by β1-integrin, we measured β1 expression in suspended keratinocytes. As shown in figure 1b, more than 90% β1-integrin transcription was suppressed after 24 h of suspension in methylcellulose. The suspension-induced differentiation increased the level of K15 as well as involucrin proteins, whereas K14 protein was not influenced significantly (Fig. 1c). This was supported by the immunofluorescence staining data shown in figure 2. Suspension of single cell N-Terts in methylcellulose induced cell aggregation, which stained much more strongly for K15, involucrin and cornifin (Fig. 2). On the other hand, the staining for K14 was not influenced by suspension.

When N-Terts were grown in the RM+ medium, containing 50-fold more EGF than the SFM, the expression profile became biphasic, an initial de-differentiation phase followed by the differentiation phase (Fig. S1a). Substitution of N-Terts with normal human primary epidermal or immortalized keratinocytes (HaCaT) in the suspension culture only changed the level of gene induction, without affecting the overall pattern of K15 induction or expression of the differentiation markers (Fig. S1b).

### Suppression of K15 Expression in Suspension Culture by Fibronectin and RGD Peptide

Engaging β1-integrin with fibronectin has been shown to suppress methylcellulose induced terminal differentiation of keratinocytes [43]. To demonstrate that K15 expression was indeed sensitive to keratinocyte differentiation, we added 100 µg/ml fibronectin in the suspension culture, which suppressed terminal differentiation as shown by the decrease in the level of cornifin mRNA (Fig. 1d). The expression of FOXM1 (isoform B), which is known to be downregulated at the onset of differentiation [44], was increased by fibronectin, confirming that differentiation was being suppressed. Interestingly, the expression of K15 was also suppressed by fibronectin (Fig. 1d), suggesting that.

K15 expression was sensitive to differentiation under these conditions. The fibronectin effect was reproduced by 5 µM arginine-glycine-aspartate (RGD) peptide, which is known to mimic fibronectin [43], [45], except that the peptide was more effective, perhaps due to its low molecular weight. As expected, the expression of K14 and FOXM1B, which are reduced in suspension, were increased by fibronectin and the peptide (Fig. 1e).

### Induction of K15 Expression at High Keratinocyte Density

**Figure 3:**
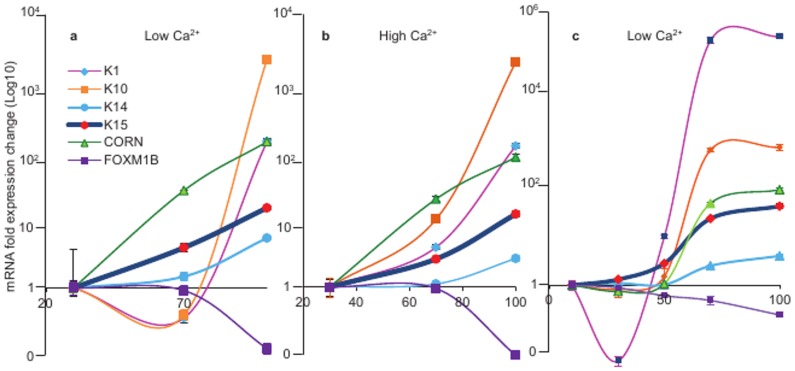
Induction of K15 transcription in keratinocytes cultured at increasing cell densities. N-Terts growing in SFM at low (0.09 mM) (a) and high (1.8 mM) (b) calcium concentrations were allowed to reach the desired confluence before being used for expression of K1, K10, K14, K15, cornifin and FOXM1B. In (c) N-Terts at low calcium (0.09 mM) were plated at the required density, allowed to attach for 24 h, after which they were used to measure mRNA levels by qPCR. Each bar represents the mean±SEM where n = 3.

Keratinocytes are reported to undergo differentiation at a high cell density [41]. To investigate how cell density-induced keratinocyte differentiation influences K15 expression, we measured K15 mRNA in N-Terts, cultured to high density. We observed that at low calcium (0.09 mM), the transcription of K10, K1 and cornifin increased 1774, 120 and 120 fold respectively, as the cell density increased from 20 to 95% (Fig. 3a). The basal keratins K14 and K15 were also induced at high cell densities, with K15 showing a much higher expression (14 fold) than K14 (5.1 fold). The FOXM1B expression was slightly reduced (about 10%) as the cell density approached confluence. This pattern remained unchanged at 1.8 mM calcium (Fig. 3b).

To investigate if increasing cell-cell interaction/adhesion was enough to trigger differentiation and K15 expression, we plated N-Terts at different densities (from 20–100%) in low calcium (0.09 mM) and after 24 h analyzed the expression of K1, K10, cornifin, K14, K15 and FOXM1B. As shown in figure 3c, the expression pattern was almost identical to that seen when the cells were allowed to grow for several days to reach confluence.

**Figure 4:**
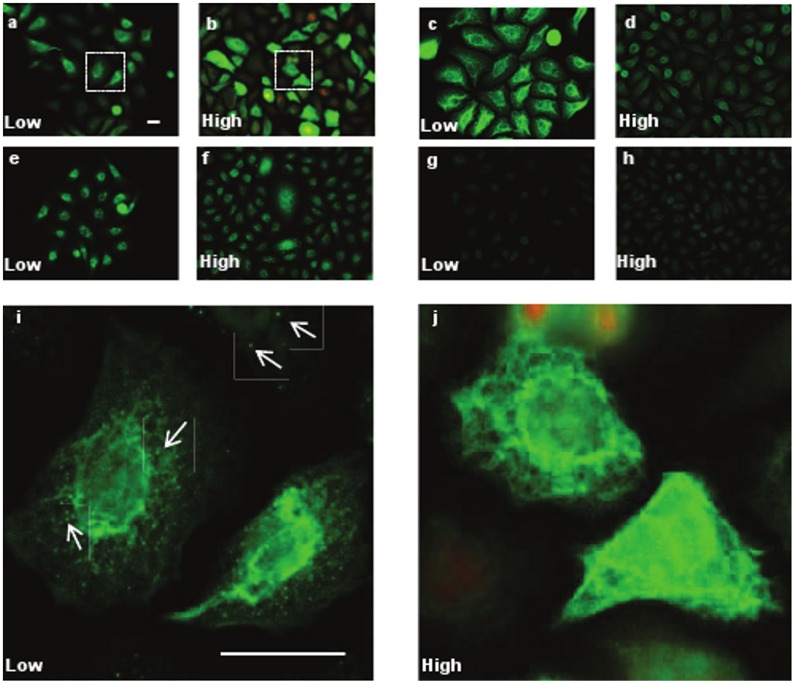
Induction of K15 at high cell density. N-Terts were grown on coverslips at low (30%) or high (95%) cell densities in RM+. The cells were fixed in formaldehyde and immunostained with antibodies against K15 (a, b), K14 (c, d), involucrin (e, f) and cornifin (g, h). All slides were photographed at the same magnification. The insets in a and b were magnified in i and j, respectively, to show the presence of K15 aggregates, shown by arrows, in keratinocytes grown at low cell density (magnification bar = 20 µm).

A comparison of protein expression in N-Terts was carried out at low and high cell densities using immunofluorescence staining. As shown in figure 4, cells at low density expressed reduced K15 (Fig. 4, compare a with b) but the expression of involucrin (Fig. 4e, f) was not significantly different at low and high cell densities. K14 expression was significantly reduced at high cell density (Fig. 4c, d). The cells at the low density expressed K15 as small globules spread in the cytoplasm whereas at the high cell density only filament staining was predominantly observed (Fig. 4i, j).

To determine if density-dependent induction of K15 was unique to N-Tert, we replaced N-Terts with a spontaneously immortalized human keratinocyte, HaCaT, in these experiments. The HaCaT cells were plated at 30% or at 95% confluence and after 24 h analyzed for the mRNA expression of K1, K10, K14, K15, cornifin and FOXM1B. The differentiation-specific markers K1 and K10 were induced 20 and 5 fold, respectively, whereas cornifin was only increased 2 fold. FOXM1B expression was not influenced, but K14 was induced 2 fold (Fig. S2a). The protein expression was increased for K14 but not significantly for K15 (Fig. S2b).

### Role of PKC in the Induction of K15 Expression

**Figure 5:**
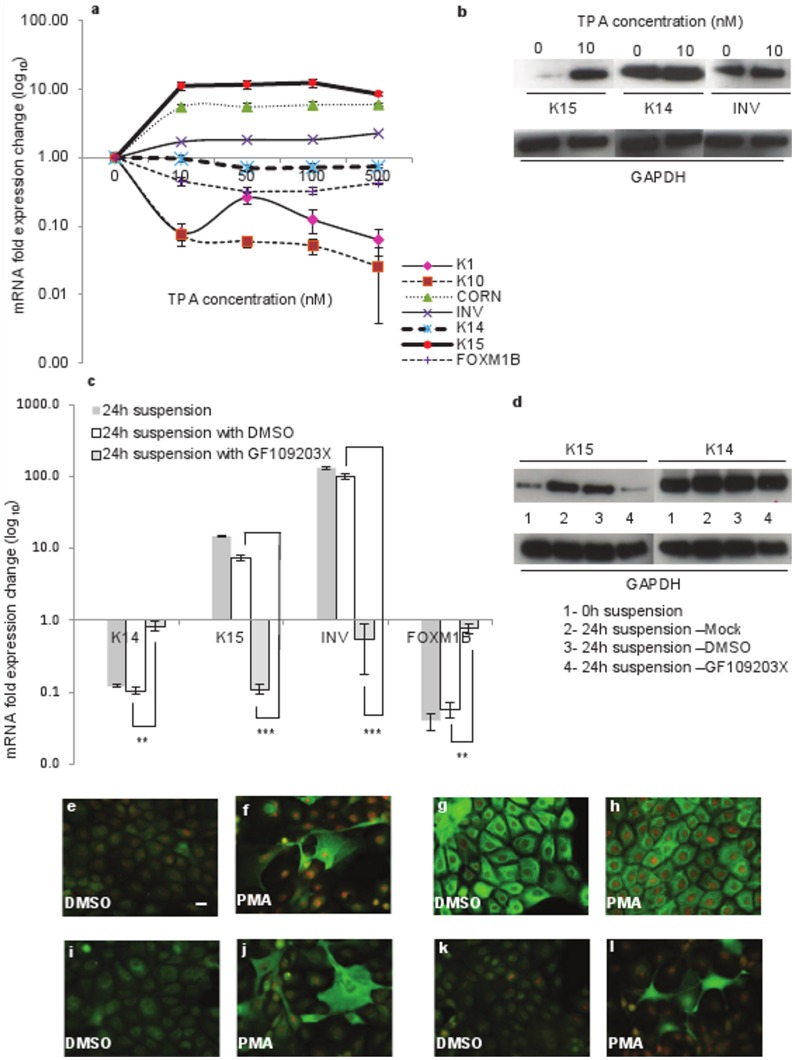
Influence of PKC activator and inhibitor on the expression of K15 in keratinocytes. (a) N-Terts in RM^+^ were exposed to different concentrations of PMA (0–500 nM) or DMSO for 24 h and mRNA expression was determined by qPCR. (b) The keratinocytes were exposed overnight to either 0 or 10 nM PMA, and protein levels were determined by western blotting. (c) N-Terts grown in SFM were suspended in 1.3% (w/v) methylcellulose with no additive, 0.02% DMSO, or 5 µM PKC inhibitor GF109203X for 24 h and the cells were used to determine mRNA expression. Freshly trypsinised single cell suspension of N-Terts was used as 0 h control cells. (d) N-Tert keratinocytes were suspended as in (c) with GF109203X and protein expression at 0 h or 24 h was determined by western blotting. Each bar represents the mean±SEM where n = 3. (P<0.01, very significant, **; P<0.001, extremely significant, ***). For immunostaining N-Terts growing on glass coverslips in RM+ were treated with 10 nM PMA (f, h, j, l) or DMSO control (e, g, i, k) for 24 h. The cells were fixed in 3.8% formaldehyde and immunostained with antibodies against K15 (e, f), K14 (g, h), involucrin (i, j) and cornifin (k, l) (magnification bar = 20 µm).

Exposure of keratinocytes to PMA has been reported to activate PKC and induce differentiation [46]. To investigate if PMA-induced differentiation activates K15, we treated N-Terts with different concentrations (0–500 nM) of PMA and observed induction of cornifin and involucrin whereas K1, K10 and FOXM1B were suppressed. The expression of K14 was not influenced, whereas K15 was induced about 10 fold at 10 nM PMA (Fig. 5a). These data were confirmed by western blotting where K15 was induced by PMA, K14 was uninfluenced and involucrin was only slightly induced (Fig. 5b). Immunostaining of N-Terts treated with 10 nM PMA showed enhanced expression of K15, involucrin and cornifin, whereas expression of K14 was only slightly reduced (Fig. 5e - l).

To show that suspension-induced K15 expression also involves PKC activation, we suspended N-Terts in methylcellulose in the presence of a highly specific PKC inhibitor GF109203X [47]. As shown in figure 5c, 5 µM GF109203X increased K14 and FOXM1B expression, whereas involucrin and K15 were massively suppressed. The expression of K15 protein was also suppressed by GF109203X but K14 was not influenced (Fig. 5d), suggesting that the inhibitor was highly selective for K15. These observations highlighted that the mechanisms regulating K14 and K15 genes were different.

### Knocking Down PKCδ but not PKCη Suppresses Suspension-induced K15 Expression

Epidermal keratinocytes express five different isoforms of PKC [48]. To determine which PKC isoform is involved in suspension-induced K15 expression, we first identified the PKC isoforms that were transcribed following suspension of N-Terts in methylcellulose. Only PKCδ and PKCη transcriptions were induced more than 2 fold and the rest did not change (Fig. S3).

**Figure 6:**
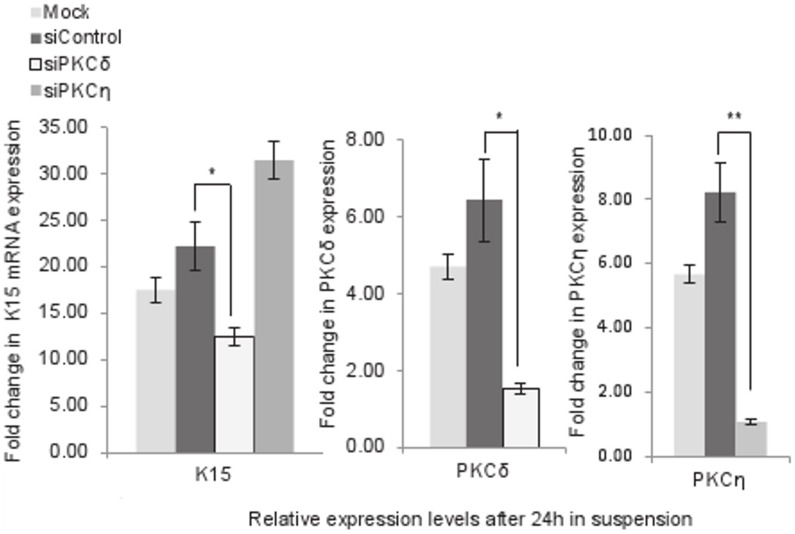
Knocking down PKCδ suppresses suspension-induced expression of K15. N-Terts were transfected separately with mock (no oligo), control siRNA or siRNA for PKCδ or PKCη. After 48 h of transfection, the cells were suspended in methylcellulose for 24 h, and used to determine the mRNA expression of K15, PKCδ and PKCη by qPCR. Each bar represents the mean±SEM where n = 3. (P<0.05, significant, *; P<0.01, very significant, **).

Using siRNA, we independently knocked-down PKCδ or PKCη to determine its effect on suspension-induced K15 expression. We found that knocking down PKCδ to about 80% reduced K15 transcription by about 50%, whereas knocking down PKCη was ineffective (Fig. 6).

### Role of AP-1 in Suspension-induced K15 Expression

**Figure 7:**
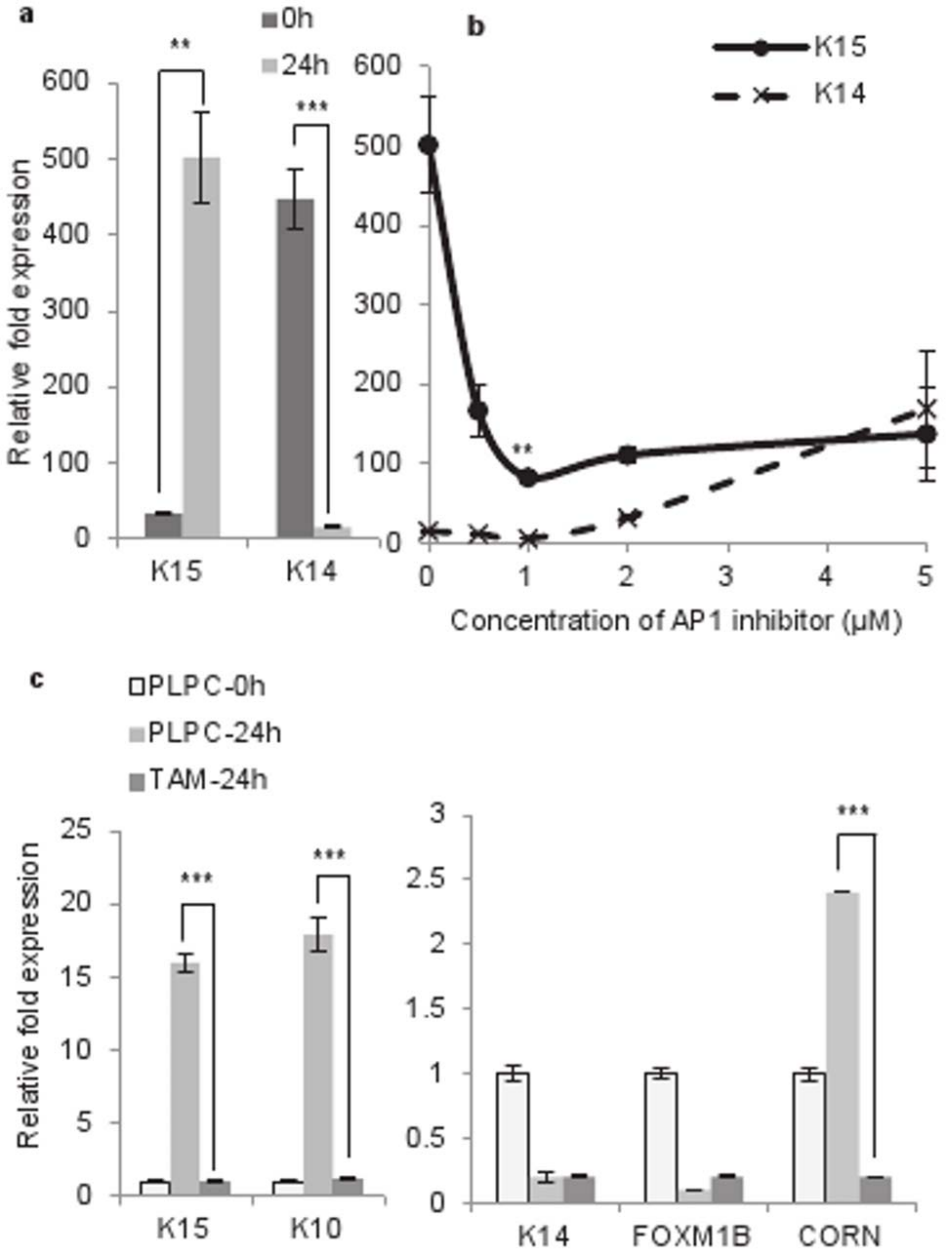
Role of AP-1 in the suspension-induced expression of K15. (a) Induction of K15 but suppression of K14 after 24 h suspension of N-Terts in methylcellulose. (b) N-Terts were treated with different concentrations of SR11302 or DMSO before being suspended in methylcellulose. After 24 h, the cells were used to determine mRNA expression of K14 and K15. (c) HaCaT cells were transduced with retroviruses expressing TAM67 or the pLPC vector control. After puromycin selection, the cells were suspended in methylcellulose for 24 h and used for gene expression analysis by qPCR. Each bar represents the mean±SEM where n = 3. (P<0.01, very significant, **; P<0.001 extremely significant, ***).

One of the mechanisms of terminal differentiation of keratinocytes involves the MAP kinase pathway that leads to induction of AP-1, a transcription factor that is a complex of a member of the Jun and Fos families of proteins [49]. To investigate whether AP-1 activation is required for K15 transcription in the suspension culture, N-Terts were treated with different concentrations of a highly specific AP-1 inhibitor SR11302 [50] followed by the methylcellulose suspension. As the concentration of the inhibitor was increased from 0 to 2 µM, the K15 transcription decreased by about 75%, without influencing K14. Further increasing the inhibitor from 2–5 µM did not change K15, but the K14 expression was increased (Fig. 7b). These experiments further highlight differences in the regulatory mechanisms of K14 and K15, although both are basal-specific type I keratin genes.

To further demonstrate the role of AP-1 in K15 expression, HaCaT cells were transduced with the retrovirus expressing TAM67, the dominant negative form of c-Jun, or pLPC vector. Expression of TAM67 significantly inhibited the suspension-induced HaCaT differentiation as shown by a reduction in cornifin expression compared with the vector control (Fig. 7c). The expression of K15 followed the same trend as cornifin, but K14 and FOXM1B were marginally influenced (Fig. 7c). This complements the data shown in figure 7b using the AP-1 inhibitor SR11302.

### Role of FOXM1B in K15 Expression

**Figure 8:**
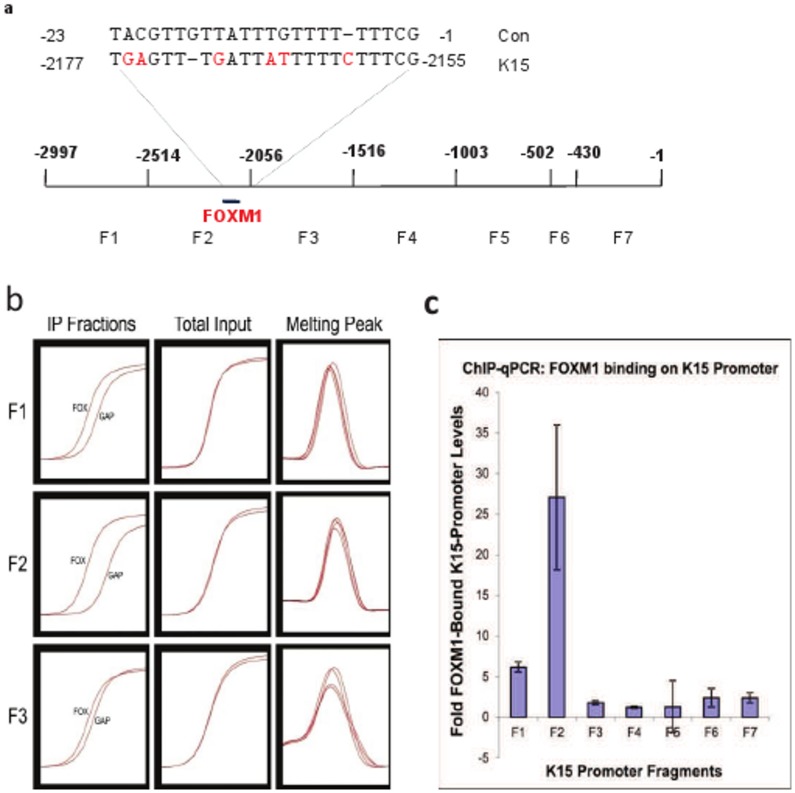
FOXM1B binding site on the K15 promoter. (a) K15 promoter divided into seven fragments (F1–F7) with the unique FOXM1B binding motif (K15) and its comparison with the consensus sequence (con) is shown and the nucleotides in red highlight the differences. (b) X-chromatin immunoprecipitation (X-ChIP): Primary human keratinocytes transduced with FOXM1B-GFP chimera were subjected to X-ChIP using FOXM1B and GAPDH (as control) antibodies. The immunoprecipitated DNA was quantified by qPCR using primers specific for different regions (F1–F7) (see panel a). All primers produced a single product (Melting peak) and the trace before (Total input) and after addition of FOXM1B (FOX) and GAPDH (GAP) antibodies (IP Fraction) is shown. Data for only 3 primer sets are shown for clarity. (c) The DNA fragments immunoprecipitated with FOXM1B antibody were quantified by qPCR. Each bar represents the mean±SEM where n = 3.

We have recently shown that the ectopic expression of FOXM1B in normal keratinocytes dose-dependently induces K15 gene transcription [44]. In order to investigate if FOXM1B binds to the K15 promoter, we performed X-chromatin immunoprecipitation (X-ChIP) using a highly specific FOXM1B antibody. The immunoprecipitated K15 promoter fragments were quantified by qPCR, using primers specific for seven different regions of the promoter (Fig. 8a; Table S1). As shown in figure 8(b, c), a single fragment from the -2500 to –2000 region immunoprecipitated up to 38 fold in excess, compared to the surrounding fragments. A careful search of the K15 promoter identified a single motif (−2177–2155) in this fragment, which had 75% sequence identity with the published binding motif for FOXM1B [51] (Fig. 8a).

## Discussion

This is the first study to show that K15 is induced when keratinocytes are differentiated by anchorage deprivation in methylcellulose, or at a high cell density or by PMA. This was unexpected because K15 expression *in vivo* is confined to the basal layer; in the epidermis the expression is mostly restricted to the rete ridges, whereas in oral and other internal epithelia, it is continuous in the basal layer [21], [52]. However, in some stratified epithelia, such as the oesophagus, K15 is normally expressed in the basal and suprabasal layers [18], [22], [39]. In addition, K15 is expressed in the suprabasal layers of varicella zoster virus (VZV) blisters (Manu Singh, unpublished observation) and in oral lichen planus [40]. Furthermore, exposure of freshly cut skin sections to thyroid hormone or interferon gamma appears to induce K15 expression in the suprabasal layers [42]. This perhaps suggests that a mechanism for differentiation-specific expression of K15 exists in stratified epithelia that can be triggered by stress. However, the reason why K15 is not expressed in the suprabasal layers of the epidermis and oral epithelia is not clear. It is conceivable that a hitherto unidentified mechanism restricts K15 expression to the basal layer.

Of all the methods used in this study, only the methylcellulose suspension culture leads to irreversible loss of proliferation with the formation of corneocytes (dead cells), a process reminiscent of the terminal differentiation in skin [8], [43], [53]. The gene expression profile in the suspension culture did show dependence on the culture conditions, for example, the biphasic nature of the profile in RM+ (Fig. S1). This could be due to high concentration of EGF in RM+, which has been shown to de-differentiate keratinocytes and also suppress K15 expression [22], [36]. With the other methods of keratinocyte differentiation used in this study - high extracellular calcium concentration, high cell densities and treatment with PMA - the differentiation is either incomplete or can be reversed, with little loss of proliferation. These differences were reflected in the gene expression profiles, for example, the differentiation-specific genes, K1, K10, cornifin and involucrin were induced in the suspension culture whereas K14, a basal cell specific (or proliferation specific) marker was suppressed (Fig. 1a). When keratinocytes were differentiated at high cell density, K14 mRNA unexpectedly increased with the increase in cell density (Fig. 3). Although the basis for this observation was not clear, it has been reported previously by others [54] that signalling generated by the high cell density had an inductive effect on K14 similar to the effect on differentiation-specific genes. Similarly, the mRNA and protein levels of K15 did not increase proportionately when keratinocytes were differentiated either by PMA or in suspension culture. For example, in the suspension culture, the mRNA levels of the differentiation markers (including K15) increased more than 100 fold, whereas increase in the protein level was only 2–3 fold (compare Fig. 1a to 1c). This could be because either the mRNA synthesised during differentiation is not translated into protein or the protein is synthesised but because it is in excess of 1∶1 ratio of type I and type II polypeptides, they cannot assemble into filaments. Any unassembled keratins are highly unstable and removed by proteolysis. However, when keratinocytes were treated with PMA, the K15 mRNA level was induced 10 fold but the protein was synthesised at a much higher level than that in suspension culture (compare Fig. 1c with Fig. 5b). Furthermore, two differentiation-specific markers K1 and K10 actually decreased by PMA (Fig. 5a), which is consistent with what has been reported previously for these genes [55], [56]. Although the molecular basis for this observation is not clear, it may be that PMA activates certain PKC isoforms, which suppress these genes, or PMA triggers another mechanism with repressive effects on the transcription of K1 and K10.

Comparing the expression of K14 with K15 in the suspension culture suggests that, although the two genes are expressed in the basal layer, K14 is suppressed, whereas K15 is induced at the onset of terminal differentiation (Fig. 1a). This is the first report that has shown that β1-integrin downregulation induces K15 transcription in the suspension culture. It can be argued that the K15 induction was due to the cell cycle arrest rather than keratinocyte differentiation. However, the presence of fibronectin in the suspension culture is known to suppress differentiation without preventing cell cycle arrest [43]. In this study, we found that fibronectin and RGD peptide suppressed K15 expression, suggesting that K15 induction in methylcellulose was due to keratinocyte differentiation rather than cell cycle arrest. There are several reports that suggest that K15 was a marker of epidermal stem cells [23], [24], [25], [26], [27], [28], [29]. Although the location of stem cells in the epidermis is uncertain; some reports say they are at the tip of the rete ridges [52], whereas others point to their location within the dermal papillae [57], [58], while recent studies suggest that they could be anywhere in the basal layer [59]. What is certain, however, is that they give rise to TA cells, which move laterally away from the stem cell zone to populate the basal layer. Porter and co-workers suggested that K15 was not a stem cell marker, but instead a marker of laterally differentiated keratinocytes [22]. However, lateral differentiation does not require downregulation of β1-integrin, which is normally expressed throughout the basal layer [60], [61], [62]. It is the vertical/terminal differentiation that is triggered by the downregulation of β1-integrin [8]. Our observation that in suspension, β1-integrins are downregulated, and K15 transcription is induced suggests that K15 may also be a marker of keratinocytes undergoing vertical/terminal differentiation under certain conditions.

Treatment of keratinocytes with PMA induces differentiation and K15 expression (Fig. 5a), which is consistent with a recent report that the PMA induced epidermal injury increases suprabasal K15 expression [33]. Furthermore, treatment of keratinocytes with the differentiating agent 2-(3,4,5-trimethoxyphenylamino)-pyrrolo[2,3-d] pyrimidine has been shown to induce K15 transcription [63]. The involvement of PKC in the transcriptional induction of K15 was demonstrated by the use of PMA and a PKC inhibitor (Fig. 5). Amongst the PKC isoforms that are expressed in keratinocytes, PKCδ, PKCη and PKCε are implicated in differentiation [64], [65], [66]. However, later studies using siRNA have shown that only PKCδ and PKCη are involved [67]. In a recent study, the two isoforms inversely influenced the expression of loricrin, a differentiation marker, PKCδ inhibited, whereas PKCη induced loricrin in differentiating keratinocytes [68]. In this study, we found that knocking down PKCδ by 80% supressed K15 transcription by about 50%, whereas knocking down PKCη was ineffective (Fig. 6). At first sight the siRNA knock down data may appear inconsistent with PKC inhibitor GF109203X (Fig. 5), which was highly potent in inhibiting K15 transcription, but the two methods are not comparable. The PKC inhibitor will inhibit the enzyme activity, and therefore will always be more potent, whereas siRNA will decrease the level of PKCδ protein by degrading its mRNA. Alternatively, it is conceivable that the high potency of the GF109203X was due to inhibition of other PKC isoforms in addition to PKCδ. The activation of PKC is known to induce AP-1 in keratinocytes (reviewed in [69]), which is consistent with our observation that the inhibition of AP-1, either by SR11302 or by dominant negative c-Jun, suppresses suspension-induced K15 expression and it is also consistent with the identification of an AP-1 binding motif in the K15 promoter [42]. An intriguing observation was the suppression of K14 transcription in the suspension culture (Fig. 1, 5). On one hand the suppression of K14 synthesis in differentiating keratinocytes is consistent with what happens *in vivo*, but it is incompatible with reports in the literature that the K14 promoter is induced by AP-1 [70]. If the suspension culture induces AP-1, as our data appears to suggest (Fig. 7), then such treatment should also increase K14 expression. It is however conceivable that the AP-1 motif in the K14 promoter, although able to biochemically respond to the AP-1 [70], does not have a functional role in K14 expression, or its effect is taken over by another de-differentiation-specific mechanism in the basal layer.

Induction of K15 protein expression at high cellular densities has been reported previously [22], [41], however, the role of transcriptional, post-transcriptional and post-translational mechanisms in the K15 induction is not known [71], [72], [73]. This is the first study where high keratinocyte density has been linked with increased K15 gene transcription. We also show for the first time that the K15 synthesised at a low cellular density exists as globules, whereas at a high cell density, the filamentous form is predominantly seen. The inability of globular K15 to integrate into pre-existing K5/K14 filaments at low cell densities could be either due to its low concentration or due to chemical modification, such as phosphorylation. Whereas on one hand, the high cell density induced keratinocyte differentiation is reportedly mediated through PKC/AP-1 activation, which explains the basis for K15 induction at high cellular density [74], [75], on the other hand keratinocyte differentiation induced by high extracellular calcium alone did not influence K15 expression (data not shown), as has been reported by others [22]. This is interesting because high calcium induced keratinocyte differentiation also involves the PKC/AP-1 pathway [76] so why doesn’t calcium induce K15 transcription? It is possible that high calcium concentrations activate a PKC isoform, which is unable to produce the correct AP-1 (in terms of constituent Fos and Jun family members) conducive to K15 induction. This hypothesis is consistent with reports that PKC activation can produce different types of AP-1 complexes with different transcriptional specificities [76], [77].

The PKC/AP-1 induced expression of K15 cannot explain the basal-specific expression of K15 because most PKC isoforms and the components of AP-1 are not expressed in the basal layer [69], [78]. The sonic hedgehog inducible transcription factor, Gli1, could be involved, as it is expressed in the basal layer and is also induced in BCCs along with K15 [22], [32], but overexpression of Gli1, either by retrovirus or by knocking down patch-1, does not induce K15, indicating that Gli1 does not directly regulate K15 (Salima Mehboobali, unpublished observations). However, there are several lines of evidence to suggest that FOXM1B, a downstream target of Gli1 [79], could regulate K15 expression in basal keratinocytes. First, FOXM1B and K15 are co-expressed in the rete-ridges [44], [52] and in the outer root sheath including the bulge [80]. Second, K15 and FOXM1B are induced in BCCs [22], [32], [79]. Third, FOXM1B is a cell cycle regulated protein and is downregulated at the onset of differentiation [81], [82]. Fourth, K15 (but not K14) is induced when FOXM1B is overexpressed in keratinocytes [44]. These reports are consistent with the identification of a FOXM1B binding site in the K15 promoter (Fig. 8). Our results provide two putative mechanisms for K15 regulation, one basal (driven by FOXM1) and another suprabasal (involving PKC/AP-1) specific. These two mechanisms may not be completely independent because PKC activation could downregulate FOXM1 at the onset of differentiation [82]. This may also indicate different functions of K15 in the basal and suprabasal compartments. In the basal layer, K15 could play a role in signalling, proliferation and in cell-cell and cell-ECM interactions. In the suprabasal layers, K15 could be associated with the extra flexibility required in high traffic areas of the body, such as the oesophagus.

FOXM1B is an oncogene that is highly expressed in most cancers, which should also induce K15 in SCCs [44], [81], [83], [84], [85]. However, most published data suggests that K15 is suppressed in SCCs [32], [33]. These apparently contradictory observations could be explained by the fact that oncogenic transformation is generally accompanied with keratinocyte activation [86], which is reportedly incompatible with K15 expression [21], [36]. It is therefore plausible that FOXM1B induced K15 expression in SCCs is subsequently silenced by keratinocyte activation.

In conclusion, this study shows that keratinocytes differentiated in methylcellulose or at a high cell density or by PMA induce K15 transcription. The suspension induced K15 expression involve downregulation of β1-integrin and could be rescued by fibronectin or RGD peptide, suggesting that K15 may be a marker of vertical/terminal differentiation that was regulated by PKC/AP-1 signalling. This would explain the basis for K15 expression in the suprabasal layers of certain stratified epithelia and under certain clinical situations. The basal expression of K15 must employ a mechanism dependent on cell proliferation rather than differentiation. We speculate that the basal cell/stem cell-specific expression of K15 in the bulge and other tissues may involve FOXM1B mediated signalling, which is supported by ectopic expression, immuno-co-localisation and identification of a unique FOXM1B binding motif in the K15 promoter. The fact that keratinocytes undergoing terminal differentiation can also express K15, as shown in this study, suggests that extreme caution should be exercised when interpreting results obtained from using K15 as a marker of stem cell zones.

## Materials and Methods

Telomerase (hTERT) and p16 immortalised normal human skin keratinocytes N/Tert-1 [87] and normal human epidermal keratinocytes derived from human neonatal foreskins (cat no. 12332-011, GIBCO Invitrogen) were cultured in serum free medium (SFM) supplemented with 30 µg/ml of bovine pituitary extract and 0.2 ng/ml of epidermal growth factor (EGF), while HaCaT (a spontaneously immortalised cell line developed from human adult skin [88]) keratinocytes were cultured in DMEM +10% (v/v) foetal calf serum (FCS) and 1% (w/v) penicillin-streptomycin. Methylcellulose 4000 cP, phorbol 12-myristate 13-acetate, PMA (Sigma Aldrich, Dorset, UK), human plasma fibronectin, RGD peptide, PKC inhibitor GF109203X (Calbiochem, Nottingham, UK), AP-1 inhibitor (E,E,Z,E)-3-Methyl-7-(4-methylphenyl)-9-(2,6,6-trimethyl-1-cyclohexen-1-yl)- 2,4,6,8-nonatetraenoic acid (SR11302) (Tocris Bioscience, UK), siRNA oligos for human PKCδ and PKCη (ON-TARGET*plus SMART*pool), DharmaFECT (Thermo Scientific, Lafayette, CO), Dynabeads mRNA DIRECT kit (Invitrogen, UK), reverse transcription kit (Promega, Southampton, UK), transfection agent TransIT-LT1 (Mirus Bio LLC, Madison, USA), DC protein assay kit (Bio-Rad, Hemel Hempstead, UK) were obtained commercially. Gene expression was quantified by real-time absolute qPCR on Light Cycler LC480 using SyBR green (Roche Diagnostics Ltd, Burgess Hill, UK). The antibodies used were: Rabbit monoclonal to K15 (EPR1614Y), mouse monoclonal to involucrin (SY5), mouse monoclonal to cornifin (SPRR3), Rabbit polyclonal to GAPDH (abcam, Cambridge, UK), anti-cytokeratin 14 LLOO2 (Cancer Research UK), FOXM1B C-20 (Santa Cruz Biotechnology, Santa Cruz, California, USA), horseradish peroxidase conjugated goat anti-mouse or goat anti-rabbit (Millipore, Watford, UK), biotin goat anti-mouse and biotin goat anti-rabbit (Sigma Aldrich), Alexa Fluor**®** 488 goat anti-mouse and Alexa Fluor**®** 488 streptavidin (Invitrogen). Western blots were detected using a chemiluminescence ECL kit (GE Healthcare, Amersham, UK).

### Induction of Keratinocyte Differentiation by Suspension in Methylcellulose

N-Terts (5×10^5^ cells) were suspended in 1.3% (w/v) methylcellulose and plated in 6 cm bacteriological dishes coated with 0.4% (w/v) poly-2-hydroxyethyl methacrylate (polyHEMA) dissolved in a mixture of acetone and 95% ethanol (1∶1). After incubating the cell suspension for the required period, it was diluted 10 fold with PBS and the cells were harvested by centrifugation. The pellet was lysed either in 500 µl of lysis/binding buffer to extract total mRNA or 200 µl of SDS sample buffer for western blotting.

In some cases N-Terts were incubated with additives (fibronectin, RGD, PKC inhibitor GF109203X, AP-1 inhibitor SR11302) before being suspended in the methylcellulose. In every incident, the influence of the effector was compared with the solvent in which the effector was dissolved. After incubation for 24 h, the cells were processed for gene expression by qPCR or for western blotting.

### Induction of Keratinocyte Differentiation at High Cell Densities and by PMA

N-Terts cultured in SFM were plated at a density of 50,000 cells in 6 cm culture dishes at low (0.09 mM) or high (1.8 mM) calcium concentrations. The cells were cultured until they reached the desired confluence and used for gene expression analysis. In some experiments however, enough cells were plated to get the desired cell density and after growing for 24 h, they were used for gene expression analysis, or lysed in SDS buffer for western blotting.

N-Terts cultured in RM+ medium were plated in 6 cm culture dishes and once attached, the cells were treated with different concentration of PMA (0–500 nM) dissolved in DMSO. The final DMSO concentration did not exceed 0.04% (v/v) in any of these experiments. After 24 h, the cells were used for gene expression and western blotting analyses.

### Use of siRNA to Knockdown PKC Isoforms in Suspension Cultures

N-Terts cultured in SFM at about 50% density were transfected with 10–100 nmol siRNA using DharmaFECT transfection reagent following the manufacturer’s instructions. The cells were allowed to grow for 48 h after which they were suspended in methylcellulose and harvested after 24 h for gene expression analysis.

### Immunofluorescence

Trypsinised N-Tert keratinocytes were seeded on sterile glass coverslips at sub-confluence or 90% confluence and allowed to attach overnight. Cells were treated with 10 nM PMA or with DMSO (final concentration less than 0.04% v/v) for 24 h. The cells were then fixed with 3.8% formaldehyde diluted in PBS for 10 min and permeabilised with 0.1% (v/v) Triton X-100 in PBS. Cells were blocked with 10% normal goat serum diluted in PBS +0.2% (v/v) Tween-20 (blocking solution) for 30 min and then incubated overnight at 4°C with primary antibodies diluted in the blocking solution. After washing three times with PBS +0.2% (v/v) Tween-20 (PBS/Tween), the cells were incubated with biotin goat anti-mouse or biotin goat anti-rabbit antibodies (depending upon the nature of the primary antibody) for 2 h at room temperature. For K14 the biotin step was omitted and the cells were directly treated with Alexa Fluor**®** 488 labelled goat anti-mouse. For the rest of the antigens, the cells were washed three times as before and incubated with Alexa Fluor**®** 488 streptavidin diluted 1∶100 in the blocking solution for 1 h at room temperature. After washing with PBS/Tween, the coverslips were treated with 5 µg/ml propidium iodide for 1 min, before being mounted using Immuno-mount and viewed in an immunofluorescence microscope using appropriate filters.

To immunostain the differentiated keratinocytes, N-Terts were suspended in methylcellulose for 24 h and harvested by dilution and centrifugation as described above. The cell pellet was suspended in 30 µl of FCS and 2–4 µl of the suspension was streaked on glass slides (SuperFrost Plus, Thermo-Scientific) using a pipette tip. Freshly trypsinised N-Terts that were not suspended in methylcellulose were used as undifferentiated 0 h control. After drying in air, the cells were fixed with formaldehyde and processed for immunofluorescence as described above.

### Gene Expression Analysis

Dynabeads mRNA DIRECT kit was used to extract the total mRNA and was immediately converted to cDNA by reverse transcription using a mixture of oligo dT and random hexamers following the manufacturer’s instructions. Absolute real time qPCR was performed for each gene as described previously [84]. Two internal reference genes (YAP1 and POLR2A) were used for target gene normalization. The qPCR primer sequences for K1, K10, K14, K15, involucrin, FOXM1B and cornifin have been described previously [44] and those for β1-integrin, PKC isoforms and K15 promoter fragments are listed in Table S1. TAM67 cDNA was PCR amplified and sub-cloned in *Eco*RI/*Bam*HI sites of pLPC_cmyc vector (Addgene, Cambridge, USA) and the construct was confirmed by sequencing. To generate replication deficient amphotropic retrovirus supernatant, Phoenix A cells were transfected with pLPC_cmyc-TAM67 construct using TransIT-LT1 following the manufacturer’s instructions. After 72 h of transfection, the cells were selected in 2.5 µg/ml puromycin until drug resistant colonies became visible. The selection was continued until the cells reached confluence. Retrovirus supernatants were collected from the confluence cultures in the absence of puromycin and stored at −80°C for future use. The transduction of keratinocytes using recombinant retrovirus supernatant was carried out using 5 µg/ml hexadimethrine bromide (polybrene) as described previously [84]. The transduced cells were selected in 2.5 µg/ml puromycin until a stable culture was established.

### Other Methods

Protein concentration was determined using the Bio-Rad DC protein assay kit. Cell counting was carried out using a haemocytometer. SDS electrophoresis was performed on 4–12% gradient gels (Invitrogen) in Bis-Tris buffer. For western blotting, the proteins were transferred electrophoretically onto nitrocellulose membranes. After probing with 1^st^ and 2^nd^ antibodies, the membranes were developed using Amersham ECL Western Blotting detection system.

### Statistical Analysis

Student’s t-tests were performed using Microsoft Excel and all results are presented as the mean of 3 individual experiments with standard error of mean (S.E.M). The P-value was calculated and was considered significant if below 0.05.

## Supporting Information

Figure S1 **Differentiation of primary human keratinocytes in suspension.** (a) N-Terts and (b) normal primary human epidermal keratinocytes growing in RM^+^ +10% FCS were suspended in DMEM +20% FCS containing 1.3% (w/v) methylcellulose. At different time intervals, cells were harvested and used to determine mRNA expression by qPCR. The RM^+^ medium contains 10 ng/ml EGF compared to 0.2 ng/ml in SFM. The initial de-differentiation phase in the graph could be due to the large excess of EGF in RM^+^. Each bar represents the mean±SEM where n = 3.
(TIF)

Figure S2 **Induction of K15 transcription in HaCaT at high cell density.** (a) HaCaT keratinocytes were grown at low (30%) and high (95%) confluence at low calcium (0.09 mM) concentration for 24 h after which the cells were lysed to determine the mRNA expression. (b) The cells grown at low (S) and high (C) densities were analyzed by western blotting for expression of K14, K15 and involucrin. Each bar represents the mean±SEM where n = 3. (P<0.01, very significant, **; P<0.001, extremely significant, ***).
(TIF)

Figure S3 **Induction of PKCδ and PKCη transcription in suspension culture.** N-Terts grown in SFM were suspended in DMEM +10% FCS containing 1.3% (w/v) methylcellulose. After 24 h the cells were harvested to determine the mRNA levels of PKCα, PKCδ, PKCη, PKCζ and PKCε by qPCR. Each bar represents the mean±SEM where n = 3. (P<0.01, very significant, **; P<0.001, extremely significant, ***).
(TIF)

Table S1 Oligonucleotides used in this study.
(TIF)
