# Supplementary figures and images for: Two Mechanisms Regulate Keratin K15 Expression In Keratinocytes: Role of PKC/AP-1 and FOXM1 Mediated Signalling
Source: PLoS One. 2012 Jun 27;7(6):e38599. doi: 10.1371/journal.pone.0038599 (PMC3384677; doi:10.1371/journal.pone.0038599)

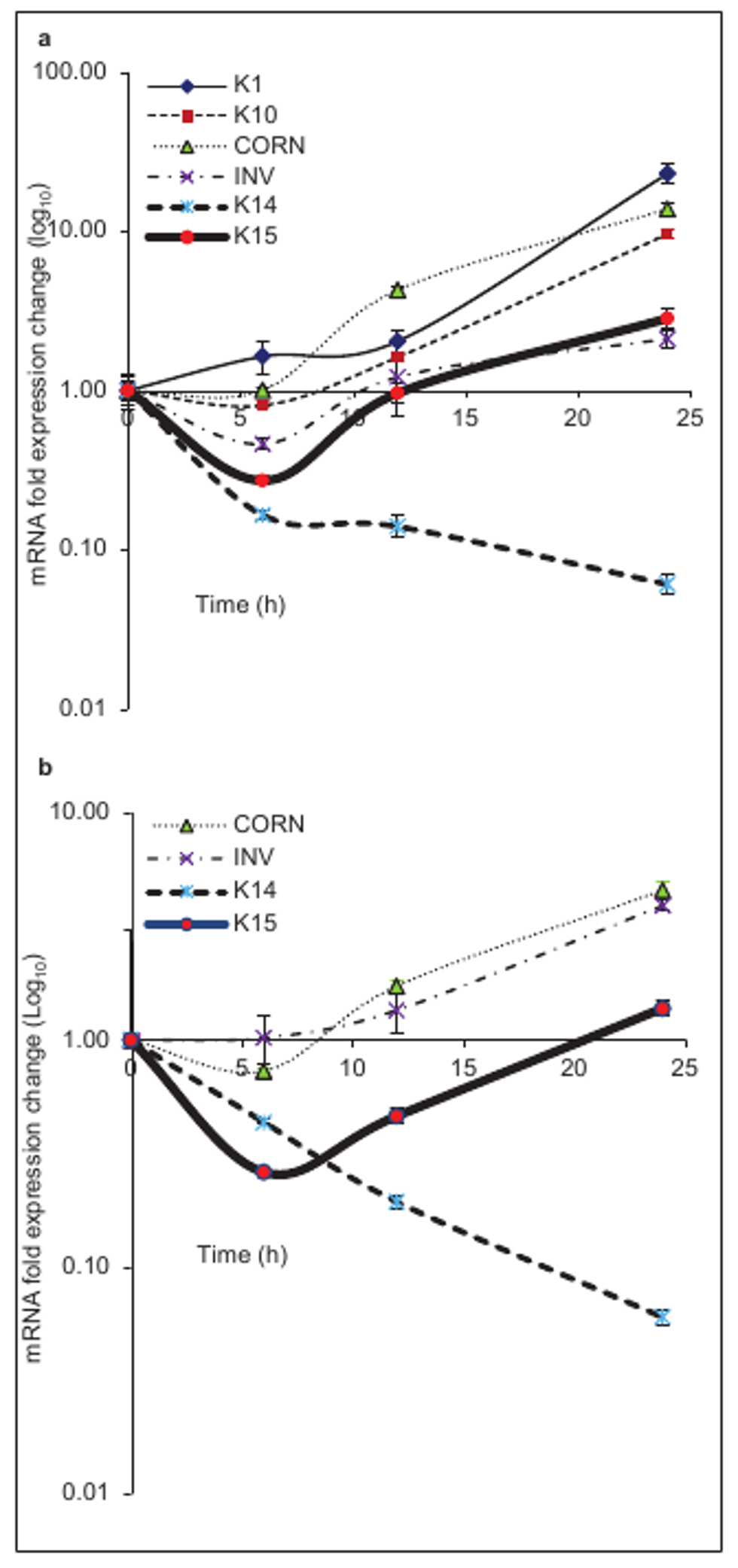

Supplement: Figure S1 — Differentiation of primary human keratinocytes in suspension. (a) N-Terts and (b) normal primary human epidermal keratinocytes growing in RM+ +10% FCS were suspended in DMEM +20% FCS containing 1.3% (w/v) methylcellulose. At different time intervals, cells were harvested and used to determine mRNA expression by qPCR. The RM+ medium contains 10 ng/ml EGF compared to 0.2 ng/ml in SFM. The initial de-differentiation phase in the graph could be due to the large excess of EGF in RM+. Each bar represents the mean±SEM where n = 3. (TIF) [file pone.0038599.s001.tif]

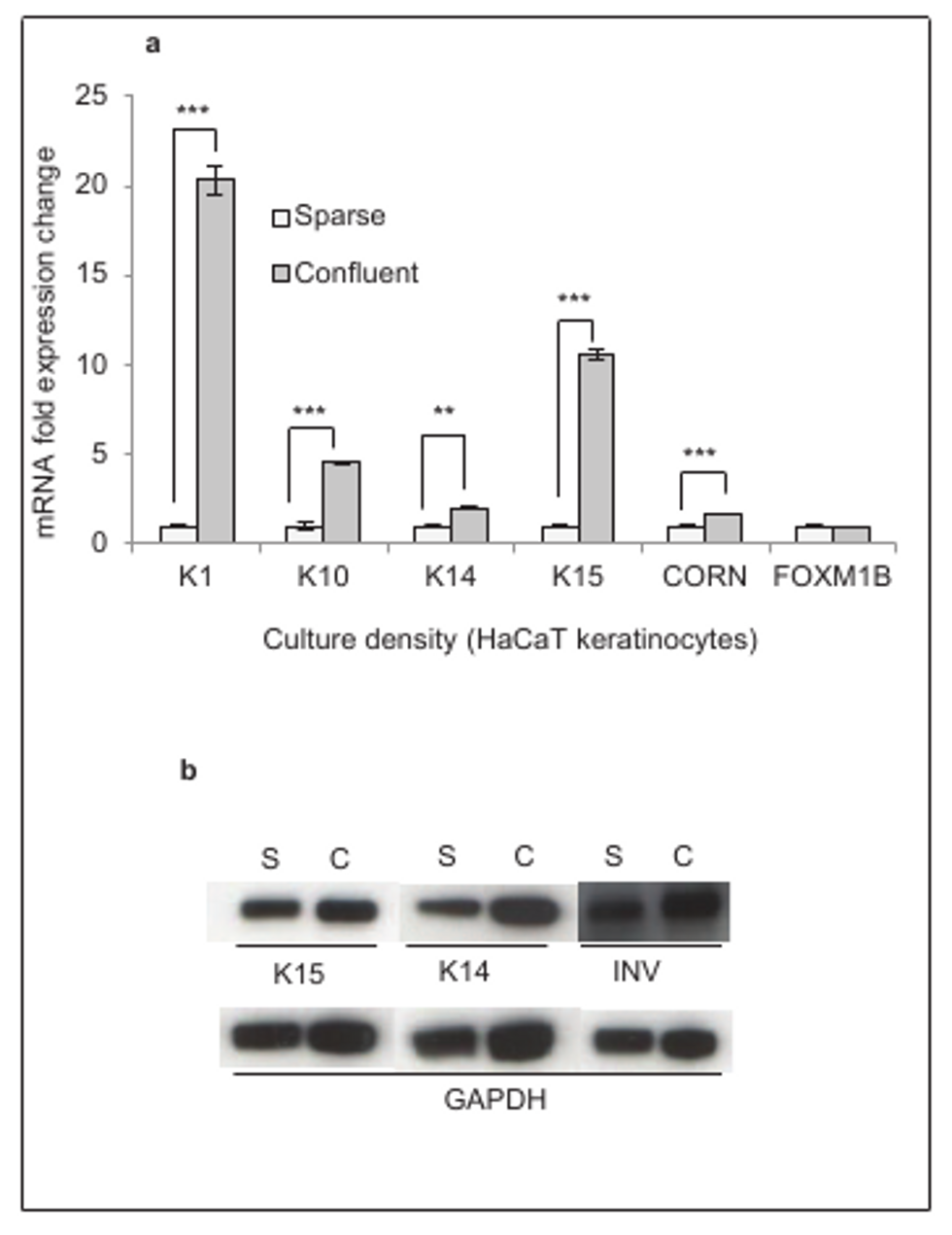

Supplement: Figure S2 — Induction of K15 transcription in HaCaT at high cell density. (a) HaCaT keratinocytes were grown at low (30%) and high (95%) confluence at low calcium (0.09 mM) concentration for 24 h after which the cells were lysed to determine the mRNA expression. (b) The cells grown at low (S) and high (C) densities were analyzed by western blotting for expression of K14, K15 and involucrin. Each bar represents the mean±SEM where n = 3. (P<0.01, very significant, **; P<0.001, extremely significant, ***). (TIF) [file pone.0038599.s002.tif]

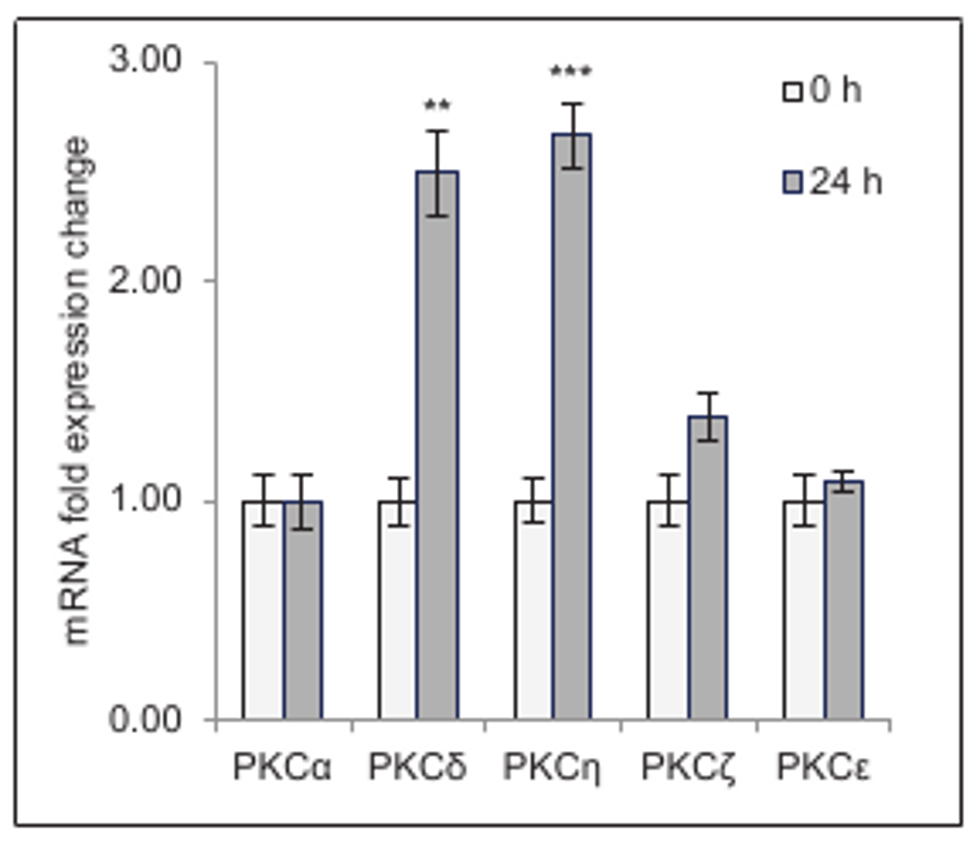

Supplement: Figure S3 — Induction of PKCδ and PKCη transcription in suspension culture. N-Terts grown in SFM were suspended in DMEM +10% FCS containing 1.3% (w/v) methylcellulose. After 24 h the cells were harvested to determine the mRNA levels of PKCα, PKCδ, PKCη, PKCζ and PKCε by qPCR. Each bar represents the mean±SEM where n = 3. (P<0.01, very significant, **; P<0.001, extremely significant, ***). (TIF) [file pone.0038599.s003.tif]
