# Supplementary material for: Two Mechanisms Regulate Keratin K15 Expression In Keratinocytes: Role of PKC/AP-1 and FOXM1 Mediated Signalling
Source: PLoS One. 2012 Jun 27;7(6):e38599. doi: 10.1371/journal.pone.0038599 (PMC3384677; doi:10.1371/journal.pone.0038599)
Supplement: Table S1 — Oligonucleotides used in this study. (TIF) [file pone.0038599.s004.tif]

Table S1: Oligonucleotides used in this study.

| **PCR primers for cloning** | | | |
| --- | --- | --- | --- |
| **Gene** | **Sequence, 5’—3’** | | **Orientation** |
| TAM67^**^ | ACGGAC***GAATTC***ATGACTAGCCAGAACACGCTGCCCA | | Forward |
|  | GCCAAT***GGATCC***TCAAAATGTTTGCAACTGCTGCGTTAGCAT | | Reverse |
| **qPCR primers** | | | |
|  |  | **Size of PCR product (bp)** |  |
| PKCα | tcgactgggaaaaactggag | 74 | Forward |
|  | ctctgctcctttgccacac |  | Reverse |
| PKCδ | attatccccgctggatcac | 71 | Forward |
|  | cttggttggttccctttcaa |  | Reverse |
| PKCη | ctgtggcacgccagactat | 66 | Forward |
|  | tctactgcaggcccgtaca |  | Reverse |
| PKCε | Aacacccgtaccttacccaac | 60 | Forward |
|  | cgaaaaagaggcggtcct |  | Reverse |
| PKCζ | ccttcctggtcggattacac | 72 | Forward |
|  | cgttgacgtactcaatgacca |  | Reverse |
| β1-inregrin | CGATGCCATCATGCAAGT | 95 | Forward |
|  | AGTGAAACCCGGCATCTG |  | Reverse |
| **K15 promoter fragment qPCR Primers** | | | |
| Fragment F1 | TGCTCCAACTGAGAGTCAGG | 64 | Forward |
|  | GCCTTAGTCTACCAATCAGGAAAA |  | Reverse |
| Fragment F2 | CCTGGGCCTGAGAGTATAGAGA | 70 | Forward |
|  | ATCCCCTTCTGCACCTGAC |  | Reverse |
| Fragment F3 | CTTGCTGGCTGGCAGTTC | 61 | Forward |
|  | CTGCAGACCCCAAATTCCT |  | Reverse |
| Fragment F4 | ACTTCACCATGCTGCAACC | 61 | Forward |
|  | CCCCAGGGCTTACAGAAAC |  | Reverse |
| Fragment F5 | TGCTAAGCAGCGACATGC | 66 | Forward |
|  | GATAAGGCTGGGGCAAGTT |  | Reverse |
| Fragment F6 | TTGGCCTGAGCTGCTCTC | 63 | Forward |
|  | GCGAACTCTCATCACAGCAC |  | Reverse |
| Fragment F7 | CACATGACCCACTCAAGGTG | 67 | Forward |
|  | AGCAAAGCCAACTCCTTCTG |  | Reverse |

** The cloning sites *Eco*RI in the forward primer and *Bam*HI in the reverse primer are shown in italics.
